# Stimulus-Evoked Calcium Transients in Somatosensory Cortex Are Temporarily Inhibited by a Nearby Microhemorrhage

**DOI:** 10.1371/journal.pone.0065663

**Published:** 2013-05-28

**Authors:** Flor A. Cianchetti, Dong Hwan Kim, Sally Dimiduk, Nozomi Nishimura, Chris B. Schaffer

**Affiliations:** Department of Biomedical Engineering, Cornell University, Ithaca, New York, United States of America; Massachusetts General Hospital/Harvard Medical School, United States of America

## Abstract

Although microhemorrhages are common in the brain of the elderly, the direct impact of these lesions on neural function remains unclear. In this work, we used femtosecond laser irradiation to rupture the wall of single arterioles in the brain of anesthetized rodents, producing a hematoma of ∼100-µm diameter. Our objective was to study the impact of these microhemorrhages on cortical activity using cell-resolved two-photon imaging of bulk-loaded calcium-sensitive dye. We monitored peripheral sensory stimulus-induced calcium transients from individual neuronal cell bodies, regions of neuropil, and astrocytes at different distances from the microhemorrhage before and 0.5, 2, and 4 hours after the creation of the lesion. We found that immediately after the hemorrhage the average amplitude of the stimulus-induced calcium response was reduced to about half within 150 µm from the hematoma. Beyond 300 µm, there was little effect on cell response, with a smooth increase in response amplitude from 150 µm to 300 µm from the lesion. Cortical function gradually improved with time and by four hours after the lesion the response from neurons and astrocytes had recovered to baseline everywhere but within 150 µm from the hematoma. To assess whether the cells closest to the microhemorrhage recovered over a longer timeframe, we developed a re-openable chronic cranial window preparation that allowed reinjection of calcium-sensitive fluorescent dye. We found that the response largely recovered by one day after the microhemorrhage even within 150 µm from the hematoma. This work suggests that neuronal and astrocyte function is transiently lost near a microhemorrhage, but recovers within one day after the lesion.

## Introduction

Small, acutely asymptomatic, hemorrhages are frequently found in the brain of elderly patients [1], [2]. These lesions range in size from several millimeters in diameter, which are observable in MRI studies [3] and often called microbleeds, to even smaller microhemorrhages of only a few tens of micrometers diameter, which are only revealed by post-mortem histology [4], [5]. The presence of even the smallest of these lesions has been associated with cognitive decline and dementia [4]–[9]. While larger hemorrhages can lead to devastating effects, the consequences of microhemorrhages remain unclear and the question of whether such lesions have direct effects on cognition via altered neuronal activity is unanswered.

It has been difficult to study the pathological consequences of a microhemorrhage due to the lack of good animal models of small vessel bleeds. Current experimental models of hemorrhage involve injection of either blood directly into the brain or enzymes that degrade the extracellular matrix, causing bleeding from many vessels [10]. Neural death and inflammation was found in the vicinity of these hemorrhages, however the hematomas created are greater than 1 mm in diameter and the mechanisms that cause cell injury after these lesions may not play the same role in microhemorrhages. Transgenic mice, such as hypertensive or CADASIL (Notch 3 mutation) animals, develop microhemorrhages with diameters of several hundred micrometers throughout the brain, which have been shown to trigger inflammation [11], [12]. However it is difficult to follow the effects of microhemorrhages on cells in these animals because of the unpredictable timing and location of the lesions. Finally, extravasation of blood plasma into the brain due to a nearby ischemic lesion did not cause dendrite degeneration [13].

Recently developed methods in *in vivo* imaging and the introduction of femtosecond laser ablation-induced models of microvascular hemorrhage have enabled studies of the impact of these lesions on the brain [14]. In recent work, we found that microhemorrhages compress surrounding tissue and cause rapid activation of microglia, leading to an increase in microglial density around the small hematoma that remains for weeks [15]. However, these microhemorrhages did not cause deterioration of nearby dendritic processes, suggesting that any effect of microhemorrhages on cognition likely comes from a process that cannot be discerned from merely structural information. While this previous work showed that neurons near the microhemorrhage did not die, the function of these cells was not characterized.

In this work, we aim to investigate changes in cortical function after a single microhemorrhage by using calcium imaging to measure neuron, neuropil and astrocyte activity. We used femtosecond laser pulses to rupture targeted arterioles in the brain of live, anesthetized rodents, creating microhemorrhages of ∼100-µm diameter [14]. We used two-photon excited fluorescence (2PEF) microscopy of bulk-loaded calcium-sensitive fluorescent dyes [16] to monitor the resulting changes in response to a peripheral stimulus. We found a decrease in neural and astrocyte response in the vicinity of the hematoma immediately after the bleed that recovered toward baseline over time, with regions further from the lesion recovering more quickly. These results show that even the smallest microhemorrhages can lead to transient loss of cortical function, but suggest that any long-term impact is not caused by the inability of neurons or astrocytes to respond to input.

## Methods

### Ethics statement

All animal procedures were reviewed and approved by the Cornell University Institutional Care and Use Committee (protocol numbers 2006-0044 and 2009-0043) and were conducted in strict accordance with the recommendations in the Guide for the Care and Use of Laboratory Animals (published by NIH).

### Acute rat preparation

We used 23 adult male Sprague-Dawley rats (Harlan, Inc.), ranging from 250 to 415 g in weight. Rats were anesthetized using 5% isoflurane (Vet-one, Inc.) and maintained at 1–2% throughout surgery. An intramuscular injection of glycopyrrolate (0.5 mg/kg rat) (Baxter, Inc.) was administered, the head was shaved, and eye ointment applied. The rat was transferred to a custom stereotaxic frame where bupivacaine (0.1 mL, 0.125%) (Hospira, Inc.) was administered subcutaneously on top of the head to provide local anesthesia. A ∼1.5-mm radius circular craniotomy was performed and the dura was removed. In order to access the hind paw somatosensory cortex, the center of the craniotomy was located 2 mm lateral and 1 mm caudal from bregma. After loading fluorescent dyes into the brain (see below), the craniotomy was filled with 1% agarose (A9793, Sigma) in artificial cerebrospinal fluid (ACSF) [17] and then sealed with a glass cover slip (50201, World Precision Instruments) that was glued to the skull using cyanoacrylate and dental cement (Co-Oral-lte Dental Mfg Corp.). One hour prior to *in vivo* 2PEF imaging, isoflurane anesthesia was gradually decreased to zero, while urethane (U25000, Sigma; diluted in deionized water) was administered intraperitonealy over three injections (final dose: 1.5 g/kg rat). Urethane was used during our studies of changes in stimulus-induced neural response after microhemorrhage because it has been reported to both maintain regulation of cerebral blood flow[18] as well as preserve the neural response to a peripheral stimulus[19]. Throughout surgery and imaging animals breathed oxygen-enriched air, body temperature was maintained at 37.5°C by a thermostatically regulated heating pad (Harvard Apparatus, Inc.), the blood oxygen saturation and heart rate were monitored using a pulse oximeter (MouseOx, Starr Life Sciences Corp.), and subcutaneous injections of 5% (w/v) glucose in physiological saline (1 ml/kg rat) were applied every hour. Animals were sacrificed at the conclusion of the imaging session.

### Chronic mouse preparation with reopenable cranial window

We used 10 male adult C57BL/6 mice ranging from 17 to 32 g in weight. Animals were anesthetized on isoflurane and eye ointment was applied. Next, intramuscular injections of glycopyrrolate (0.5 mg/kg mouse) and subcutaneous injections of dexamethasone sodium phosphate (0.2 mg/kg mouse) (American Regent, Inc.) and ketoprofen (5 mg/kg mouse) (Fort Dodge) were given. The head was shaved and cleaned with providone iodine and ethanol. After giving local injections of bupivacaine, a ∼1.5 mm radius circular craniotomy was prepared over the hind paw somatosensory cortex (center located at 1 mm lateral and 0.5 mm caudal from bregma). The dura was left intact. During all procedures when the mouse was under anesthesia, body temperature was maintained at 37.5°C by a thermostatically regulated heating pad and 0.1 mL subcutaneous injections of 5% (w/v) glucose in physiological saline were applied every hour. After loading fluorescent dyes into the brain, a thin ring-shaped wall of silicone (Kwik-sil, World Precision Instruments, Inc.) was carefully applied around the edge of the craniotomy and then over the exposed mouse skull. The craniotomy was filled with sterile ACSF and a 5-mm glass cover slip was glued to the silicone using cyanoacrylate and dental cement. This procedure created a chamber that can be removed while causing minimal injury to the brain by gently pulling the silicone away from the skull.

After closing the craniotomy, the animal was removed from anesthesia and allowed to recover for one hour. Animals were then re-anesthetized using isoflurane for *in vivo* 2PEF imaging and hemorrhage production. At the end of imaging session, the mouse was returned to a cage placed on a heating blanket and provided with wet food. We checked the window clarity and mouse behavior every eight hours until the next imaging session. Interruption of anesthesia for an hour between the surgery (2–3 hours) and the initial imaging session (4–6 hours) was found to improve the survival rate. On the next day, the mouse was re-anesthetized, the craniotomy was reopened, and fluorescent dyes were reapplied. The craniotomy was then filled with sterile ACSF and sealed by gluing a glass coverslip directly to the skull. After the second imaging session the animal was sacrificed.

### Fluorescent labeling of blood vessels, astrocytes, and neurons

**Figure 1:**
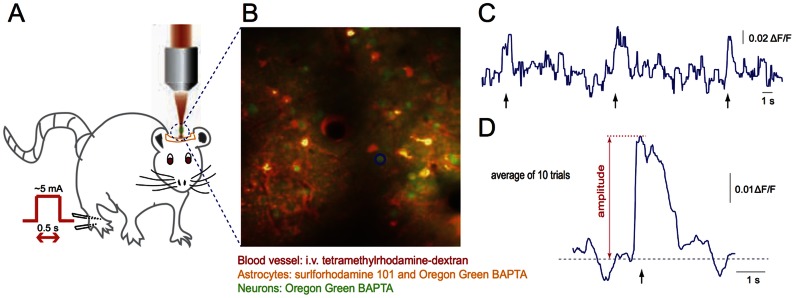
Two-photon imaging of stimulus-induced calcium transients in somatosensory cortex. **A.** Animals received an electrical stimulus to the hind paw while 2PEF microscopy was used to monitor changes in neural activity with calcium-sensitive dyes that were bulk loaded into the cortex. **B.** 2PEF image frame taken about 100 µm beneath the cortical surface in a rat. Blood vessels are labeled red (TRITC), neurons are green (OGB), and astrocytes are yellow (OGB and surlforhodamine 101). **C.** Calcium transients from the neuronal cell body circled in (B). Peripheral stimulus times are indicated by arrows. **D.** Average amplitude of the calcium transient across ten stimuli for the neuronal cell body circled in (B).

To visualize the vasculature, blood plasma was labeled via an intravenous injection of either 5% (w/v) tetramethylrhodamine isothiocyanate-dextran (TRITC) (T1287, Sigma) for rats, or 5% (w/v) Texas Red dextran (D1864; Invitrogen) for mice, each dissolved in physiological saline. We bulk loaded Oregon Green BAPTA (OGB) (06807, Invitrogen) and sulforhodamine 101 (S7635, Sigma) into the hind paw somatosensory region of cortex according to protocols described previously [16], [20]. Briefly, OGB (5 µg) was dissolved in 5-µL solution of 20% pluronic F-127 (P2443, Sigma) in dimethyl sulfoxide (DMSO) (472301,Sigma), then 40 µL of sulforhodamine 101 (50 µM) in ACSF was added. The final solution was microinjected ∼300 µm beneath the surface of the brain using a Picospritzer (III, Parker Hannifin Corp.) in three different locations throughout the hind paw representation. OGB is a calcium-sensitive fluorescent dye that labeled all brain cells. Sulforhodamine 101, a red-emitting dye that labels only astrocytes, allowed neurons and astrocytes to be distinguished (Figure 1B) [20].

### Peripheral stimulus

Electrical stimulation was applied via two 25 G needles inserted underneath the skin of the hind paw contralateral to the imaging region (Figure 1A). Ten stimulation pulses of 0.5-s duration were applied with an interval of 10 s between successive stimuli. The stimulus current (∼1 mA) was adjusted to elicit a slight movement in the toes of the paw. The average response from the 10 stimuli was used in the analysis.

### *In vivo* 2PEF imaging

*In vivo* imaging was conducted on a locally-designed 2PEF microscope using an 850-nm, 87-MHz, 100-fs pulse train from a Ti:sapphire laser system (MIRA HP; Coherent) pumped by a continuous wave laser (Verdi-V18; Coherent) for excitation. Fluorescence emission was collected in two different channels separated by a 560-nm long-pass dichroic. When imaging rats, emission filters (Chroma, Inc.) transmitting wavelengths between 485 to 550 nm and 570 to 620 nm were used to collect green (OGB) and red fluorescence (sulforhodamine 101 and TRITC), respectively. For mouse imaging, the long wavelength filter was replaced by one that transmits from 610 to 680 nm to visualize sulforhodamine 101 and Texas Red. Although imaged on the same fluorescence channel, blood vessels and astrocytes were easily distinguished based on morphology and location. Laser scanning and data acquisition was controlled by MPScope software [21].

To create a map of the cortical vasculature and to localize the regions labeled by the dye injections, we acquired a stack of images of the entire cranial window (100 µm depth) using a 0.28 numerical aperture (NA), 4X-magnification air objective (Olympus, Inc.). We changed to a 1.0-NA, 20X-magnification water-immersion objective (Zeiss, Inc.) for high-resolution imaging. We acquired 256×256 pixel frames at a rate of 13 Hz when monitoring calcium transients and 512×512 pixel frames at a rate of 3.3 Hz for image stacks.

**Figure 2:**
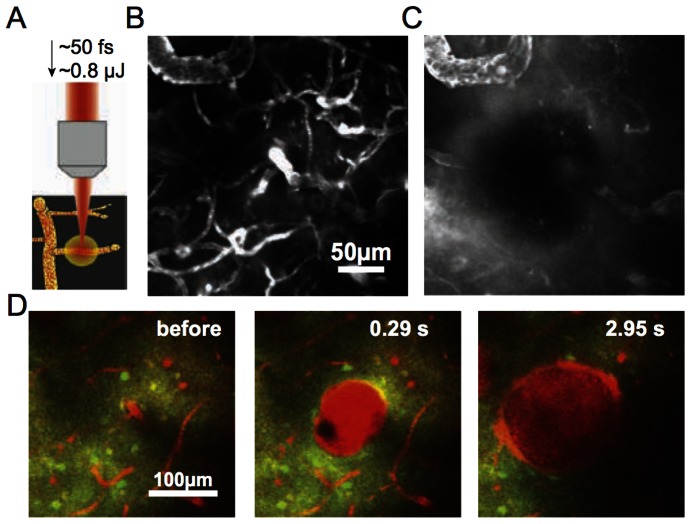
Induction of microhemorrhage with femtosecond laser irradiation. **A.** High energy, femtosecond laser pulses were tightly focused on the wall of the target vessel to rupture the vessel and initiate bleeding. **B.** Projection of 150-µm deep 2PEF image stack of fluorescently-labeled cortical blood vessels. **C.** Projection of a 2PEF image stack over the same volume as (B) after a hemorrhage was produced by rupturing the arteriole in the center of the frame. The RBC-filled hematoma core (in black) obstructed imaging, and fluorescently-labeled blood plasma was pushed into the surrounding parenchymal tissue. **D.** Time course of bleeding after rupturing a vessel about 100 µm beneath the cortical surface. Fluorescently-labeled blood plasma is red, while neurons are green and astrocytes yellow (same labeling strategy as in Figure 1B).

**Figure 3:**
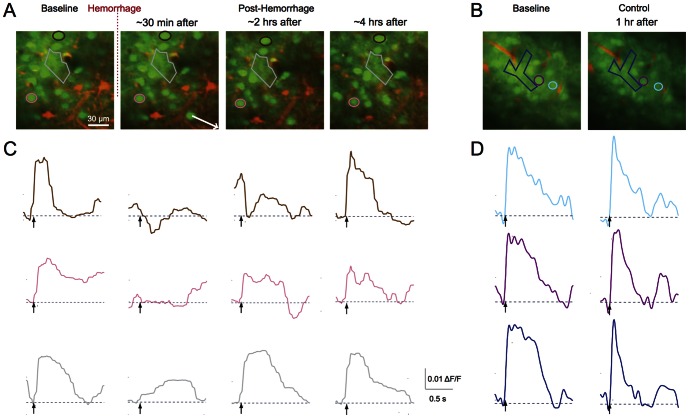
Changes in somatosensory calcium responses in neuronal cell bodies and regions of neuropil after a microhemorrhage. **A.** 2PEF images of cortex (120 µm beneath brain surface; same labeling as Figure 1B) before and over time after a microhemorrhage. The arrow in the second panel indicates the direction to the microhemorrhage, located 250 µm away. **B.** Images from a control experiment. **C.** Stimulus-induced calcium responses from two neuronal cell bodies and one region of neuropil (indicated by color coded regions in (A)) before and over time after a microhemorrhage. **D.** Calcium responses from a control experiment.

After selecting the target vessel to be ruptured (see below), we took a high-resolution stack of images centered at the hemorrhage site (Figure 2B). Then we selected between seven and ten non-overlapping 150 µm×150 µm sites that contained OGB-labeled neurons and astrocytes, located at different distances from the hemorrhage site and at a depth of 50 to 200 µm beneath the brain surface. We monitored stimulus-induced calcium transients at each of these 150 µm×150 µm locations (Figure 3). We then induced a hemorrhage in the target vessel and repeated both the high-resolution image stack (Figure 2C) and the calcium transient measurements at each site (∼30 minutes after the lesion, Figure 3). In some rats, we repeated the measurement of stimulus-induced calcium transients at two and four hours after the microhemorrhage (Figure 3). For the chronic experiments using mice we repeated these measurements one day after the lesion.

### Production of targeted microhemorrhages

To induce microhemorrhages, we injured the vessel wall of targeted penetrating arterioles using high intensity laser pulses (800-nm, 1-kHz, 50-fs pulse train from a Ti:sapphire regenerative amplifier (Legend 1 k USP; Coherent) pumped by a Q-switched laser (Evolution 15; Coherent) and seeded by a Ti:sapphire oscillator (ChinhookTi:sapphire laser; Kapteyn-Murnane Laboratories Inc; pumped by Verdi-V6; Coherent, Inc)). We focused these pulses onto the vessel wall of penetrating arterioles between 100 and 200 µm beneath the brain surface (Figure 2A). To induce a microhemorrhage, we first delivered 1 to 5 bursts of 100 pulses from the 1-kHz pulse train with an initial energy estimated to be below the threshold for causing vessel rupture (∼100 nJ at about 100-*µ*m depth)[14]. The pulse energy was then gradually increased, in 25% increments, until this irradiation (typically ∼500 nJ) lead to sufficient vessel wall damage to cause vessel rupture, allowing blood to push radially out into the brain (Figure 2D). The vessel wall clotted within seconds after the rupture, stopping the bleeding. The size of the red blood cell (RBC) filled hematoma (distinguished by dark cell-sized patches that pushed into the brain and excluded the blood plasma label) was quantified from image stacks taken within 15 minutes of producing the hemorrhage. In addition, in the mice we quantified the size of the region where fluorescently-labeled blood plasma penetrated into the brain. This was more difficult to quantify in rats because the TRITC used to label the blood plasma in these animals was not bright enough to reliably determine the limit of blood plasma penetration. One microhemorrhage was induced in each of fifteen rats and in seven mice. In addition, we performed five (three) control experiments in rats (mice), in which the same imaging protocol was followed but without the creation of a hemorrhage, as well as three sham experiments in rats, in which the vessel was targeted using laser pulse energies that were too low to rupture the vessel. Three extra mice were used for histological studies.

### Analysis of stimulus-evoked calcium transients

We implemented custom scripts in Matlab (The Mathworks, Inc.) that enabled us to compare the average amplitude of the calcium response of neuronal cell bodies and regions of neuropil at different time points. For each of the imaged sites, we manually selected between 5 and 12 neuronal cell bodies, between 4 and 6 regions of neuropil (each around ∼900 µm^2^ area) and between 0 and 8 astrocytes that could be identified at all time points before and after the microhemorrhage. For each stimulus, we calculated the normalized fluorescence change (dF/F_o_) (Figure 1C) over a 4-s window around the stimulus, where F_o_ was taken to be the mean of the lowest 50% of fluorescence values measured during the 10 s before the stimulus and dF was the instantaneous fluorescence, averaged across the cell body or region of neuropil, minus F_o_ [22]. This definition of F_o_ reduces the impact of slow changes in fluorescence due to power drifts or movement artifact. These responses were averaged across all ten stimuli and a moving median filter (50 samples wide) was applied (Figure 1D). We calculated the amplitude of the averaged calcium response for each cell or region at each time point. We excluded all cell bodies or regions of neuropil that did not exhibit a stimulus-evoked response at baseline from further analysis. We computed the ratio of the post-hemorrhage (or control/sham) response amplitude to the baseline response for each cell or region. Finally, we classified cells as having a normal post-hemorrhage response amplitude if this ratio was greater than the mean minus one standard deviation of the ratios of control and sham data.

### Post-mortem histology

In three male adult C57BL/6 mice, we implanted a chronic cranial window [23] and, after one week of recovery, we imaged the brain and triggered four microhemorrhages in penetrating arterioles, separated by at least 1 mm. We measured the diameters of the RBC filled hematoma and the surrounding plasma from *in vivo* images. Three days later animals were transcardially perfused with phosphate buffered saline (PBS, Sigma-Aldrich) followed by 4% (wt/vol) paraformaldehyde in PBS. The brain was extracted, soaked in first 30% then 60% (wt/vol) sucrose in PBS for 24 hours each. Fiducial marks were placed with 30 G needle coated with black ink to indicate the edges of the cranial window and the location of individual microhemorrhages. The tissue was frozen and cut in 20-µm thick sections on a cryostat. Tissue sections were placed on Permafrost slides (Superfrost Plus, Fisher Sci.), rehydrated with PBS, and stained with diaminobenzidine (DAB) (peroxidise substrate kit, SK-4100, Vector Labs, Inc.) to label red blood cells. To identify possible apoptotic cells, terminal deoxynucleotidyl transferase dUTP nick end labeling (TUNEL) was completed (11684795910, Roche Applied Scientific, Inc.). Briefly, sections were permeabilized in a 0.1% (wt/vol) Triton X-100, 0.1% (wt/vol) sodium citrate solution in a 4°C refrigerator for 5 min., then washed in PBS twice for 2 min. each, and treated with the TUNEL enzyme/labeling solution. The tissue sections were covered with aluminum foil to exclude light and incubated at 37°C for 3 hours. Sections were then rinsed in PBS twice for 3 min. each and imaged with an epifluorescence microscope. We checked for TUNEL-positive cells in sections that contained a microhemorrhage, as identified both by the location relative to the fiducial marks and the presence of DAB-positive red blood cells in the tissue.

### Statistical Analysis

**Figure 4:**
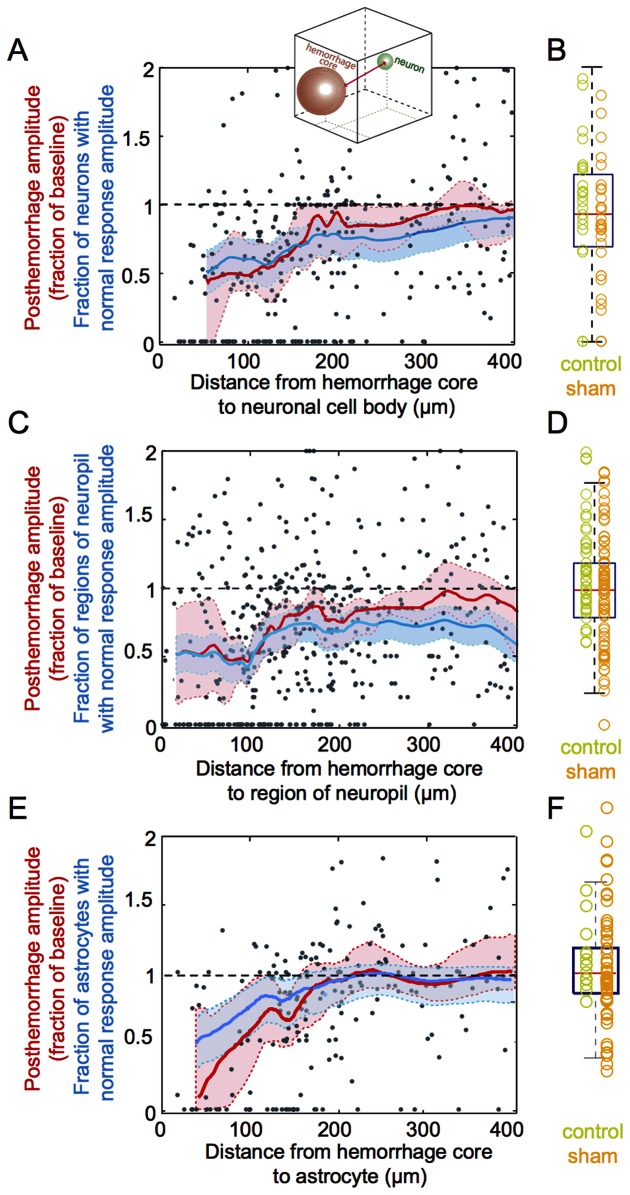
Post-hemorrhage neural response as a function of distance from the hematoma. **A.** Amplitude of the stimulus-induced calcium responses for neuronal cell bodies at different distances from the hematoma core, measured within 30 minutes of lesioning, and expressed as a fraction of the baseline responses. Grey points represent measurements from individual neuronal cell bodies rats, while the red line indicates a moving median of the response amplitude. The blue line represents the fraction of neuronal cell bodies that continued to respond normally to the peripheral stimulus after the microhemorrhage (i.e. with a response amplitude ratio that was greater than the mean minus one standard deviation of the amplitude ratio for control and sham data). The red and blue shaded regions represent 95% confidence intervals about the median response amplitude and fraction of cells normally responding, respectively. Distance is defined as the three-dimensional path from the edge of the spherical RBC-filled hematoma core to the center of the neuronal cell body (inset). **B.** Box plot of the amplitude of the calcium response from neuronal cell bodies expressed as a fraction of the baseline response, for control and sham experiments. The measurements from individual cells are indicated with green (control) and orange (sham) circles. **C.** Amplitude of the stimulus-induced calcium responses for regions of neuropil at different distances from the hematoma core, within 30 minutes of lesioning, and expressed as a fraction of the baseline responses. **D.** Box plot of the amplitude of the calcium response from regions of neuropil, expressed as a fraction of the baseline response, for control and sham experiments. **E.** Amplitude of the stimulus-induced calcium responses for astrocytes at different distances from the hematoma core, within 30 minutes of lesioning, and expressed as a fraction of the baseline responses. **F.** Box plot of the amplitude of the calcium response from astrocytes, expressed as a fraction of the baseline response, for control and sham experiments. Data was taken across 15, 5 and 3 rats for hemorrhage, control and sham experiments, respectively.

Moving medians of post-hemorrhage response amplitudes and the 95% confidence interval was obtained as a function of distance from the microhemorrhage using a moving window size of 1/10th of the total number of available data points: 35, 45, and 30 samples for neuronal cell bodies (Figure 4A), regions of neuropil (Figure 4C), and astrocytes (Figure 4E), respectively. The plots were further smoothed using a 20-sample moving average. Boxplots containing both control and sham data were produced for neurons (Figure 4B), neuropil (Figure 4D), and astrocytes (Figure 4F), respectively. The edges of the box represent the first and third quartile of the data, the red line across the box indicates the median and the whiskers extend to the maximum and minimum amplitude values that are not outliers. Individual data points are shown in green for control and in yellow for sham experiments (Figure 4B, Figure 4D and Figure 4F).

**Figure 5:**
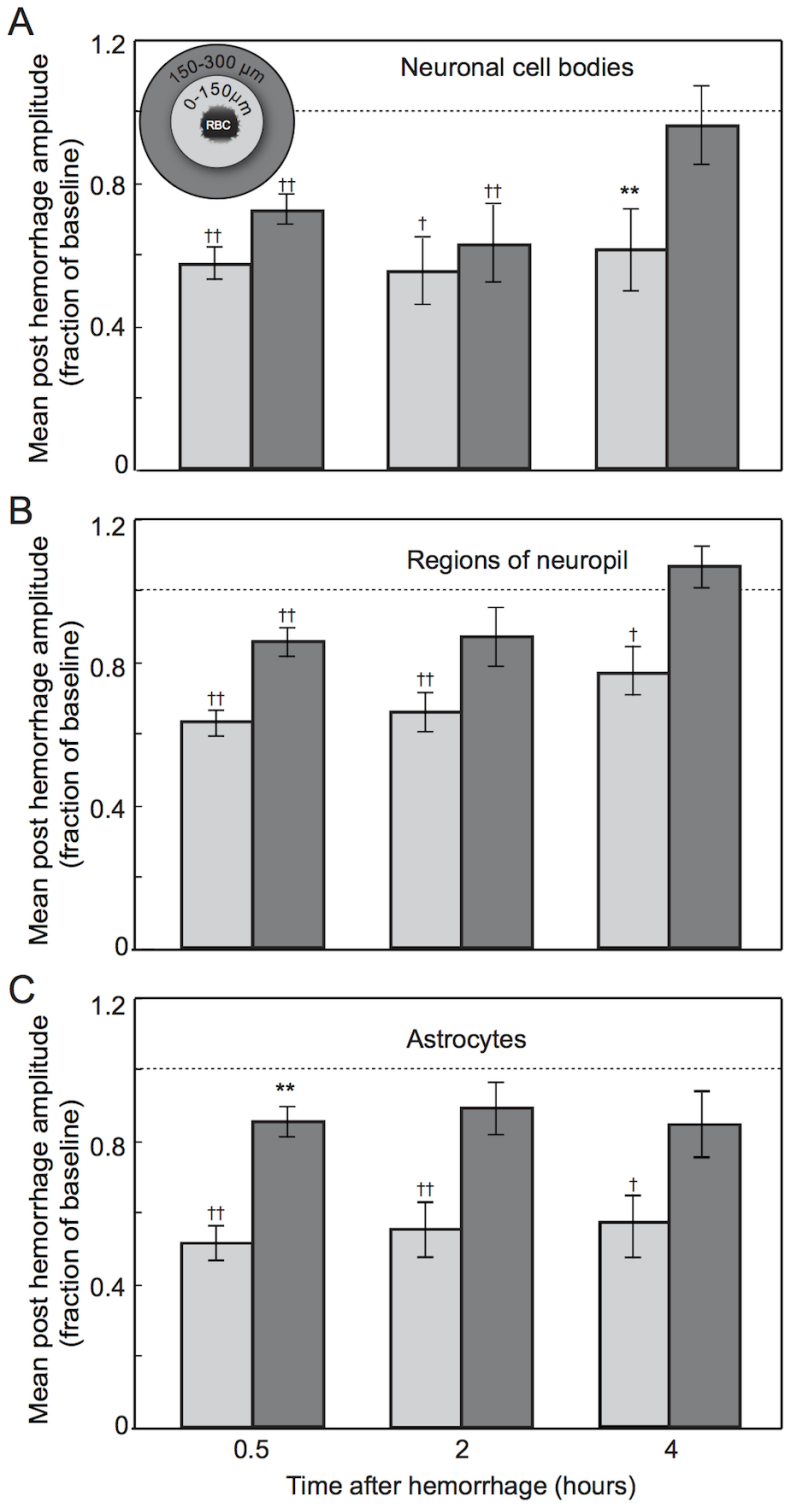
Changes in average neuronal response over hours after a microhemorrhage. **A.** Average of the post-hemorrhage stimulus-induced calcium response amplitudes expressed as a fraction of the baseline response amplitudes, for neuronal cell bodies at 0.5, 2, and 4 hours after a microhemorrhage and for cells located either within 150 µm (light grey) or between 150 and 300 µm (dark grey) of the RBC-filled hematoma, as indicated in the inset. Error bars represent the standard error of the mean. Dashed line indicates a response amplitude after the microhemorrhage that is equal to the baseline response. **B.** Average response for regions of neuropil. **C.** Average response for astrocytes. Levels of significance for differences of the average amplitude ratios from one are indicated by: * p<0.05, ** p<0.01, † p<0.001, †† p<0.0001 (Wilcoxon signed-rank test). Data was taken across 15 rats.

Bar plots were used to compare the average post-hemorrhage response amplitude ratios at different times after the lesion and at different distances from the microhemorrhage (Figure 5). Error bars represent the standard errors of mean. For these data, distributions were found to be non-normal (Shapiro-Wilk W test), so we used rank-based statistical analysis. We performed Wilcoxon signed-rank tests to determine if the response amplitude ratios were less than one.

## Results

We investigated the response of somatosensory neuronal cell bodies, regions of neuropil, and astrocytes to a peripheral stimulus using 2PEF imaging of calcium sensitive dyes (Figure 1) before and after femtosecond laser-induced hemorrhage of a nearby microvessel (Figure 2). The microhemorrhage consisted of a RBC-filled hematoma core of about 100+/−40 µm (mean +/− standard deviation) diameter (15 hemorrhages in 15 rats; 19 hemorrhages in 10 mice). Surrounding this core was a 280+/−110 µm diameter region where fluorescently-labeled blood plasma pushed into the brain parenchyma (Figure 2). The targeted vessel was observed to be flowing after each hemorrhage. For 15 rats and 7 mice we characterized the changes in stimulus-evoked neural activity as a function of distance from the microhemorrhage and as a function of time after the lesion (Figure 3), up to one day later.

### Average response amplitude to a peripheral stimulus decreased immediately after a microhemorrhage in neurons located in nearby areas

We found that within 30 minutes after a microhemorrhage in the acute rat preparation, the amplitude of the calcium response either disappeared or decreased in most of the neuronal cell bodies, regions of neuropil, and astrocytes located within 150 µm of the edge of the RBC-filled core of the microhemorrhage (Figure 3 and Figure 4). In this region, the average ratio of the response amplitude to the baseline response was 0.6+/−0.5 (mean +/− standard deviation), 0.6+/−0.6, and 0.5+/−0.4 for neuronal cell bodies (Figure 4A), regions of neuropil (Figure 4C), and astrocytes (Figure 4E), respectively. Both control and sham experiments showed no decrease in the average response amplitude of neuronal cell bodies (Figure 4B), regions of neuropil (Figure 4D), and astrocytes (Figure 4F). Further from the microhemorrhage the immediate impact of the lesion on stimulus-evoked response amplitude was more modest, with only a minor decrease in activity from 150 to 300 µm from the RBC-filled hemorrhage core and no decrease in activity further than 300 µm (Figure 4A, Figure 4C and Figure 4E). Further, we determined the fraction of neuronal cell bodies, regions of neuropil, and astrocytes with a stimulus-evoked response amplitude that was similar to the response amplitude in controls (i.e. the post-hemorrhage response was greater than one standard deviation below the mean response in controls). We found that the fraction of neuronal cell bodies, regions of neuropil, and astrocytes with normal response amplitude was lowest near the microhemorrhage and recovered with distance from the microhemorrhage with a similar trend to the post-hemorrhage response amplitudes (Figure 4A, Figure 4C, and Figure 4E).

### The response to a peripheral stimulus recovered toward baseline with time after the microhemorrhage

Over time, we observed a distance-dependent recovery of function in the neuronal cell bodies, regions of neuropil, and astrocytes that showed a decreased response immediately after the microhemorrhage. In acute rat experiments, the neuron response amplitude recovered to baseline values by four hours after the microhemorrhage at distances of 150 and 300 µm from the edge of the RBC-filled hematoma. Regions of neuropil and astrocytes recovered by 2 hours. In contrast, activity remained depressed within 150 µm (Figure 5). Longer duration acute experiments were impractical due to the inability to maintain stable physiology during extended anesthesia.

**Figure 6:**
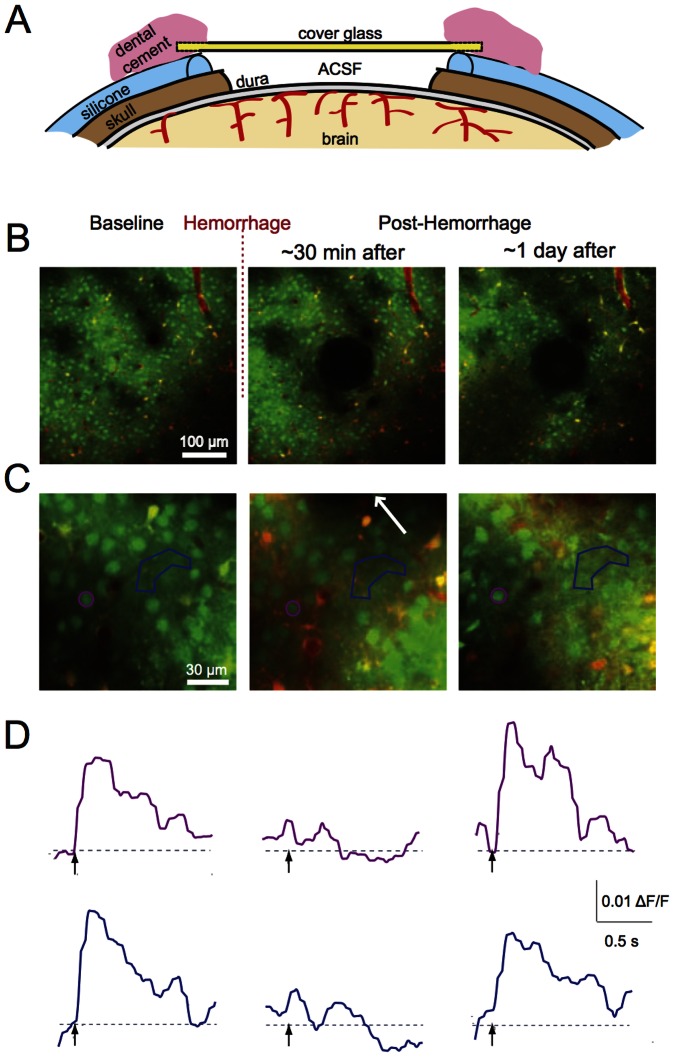
Chronic imaging of stimulus-induced calcium transients after a microhemorrhage. **A.** Schematic of a re-openable chronic cranial window preparation for mouse. A layer of silicone coated the skull around the craniotomy, and the glass was glued to the silicone. The window was reopened by gently detaching the silicone from the skull, enabling reinjection of OGB and sulforhodamine 101 into the cortex. **B.** Low- and **C.** high-magnification 2PEF images of the same regions of the brain before, immediately after, and one day after inducing a microhemorrhage. The hematoma is visible in the center of the second and third panels in (B). The arrow in the second panel of (C) indicates the direction to the microhemorrhage, located 40 µm away. **D.** Stimulus-induced calcium responses from the neuronal cell body and region of neuropil indicated on panel (C) by color coding.

**Figure 7:**
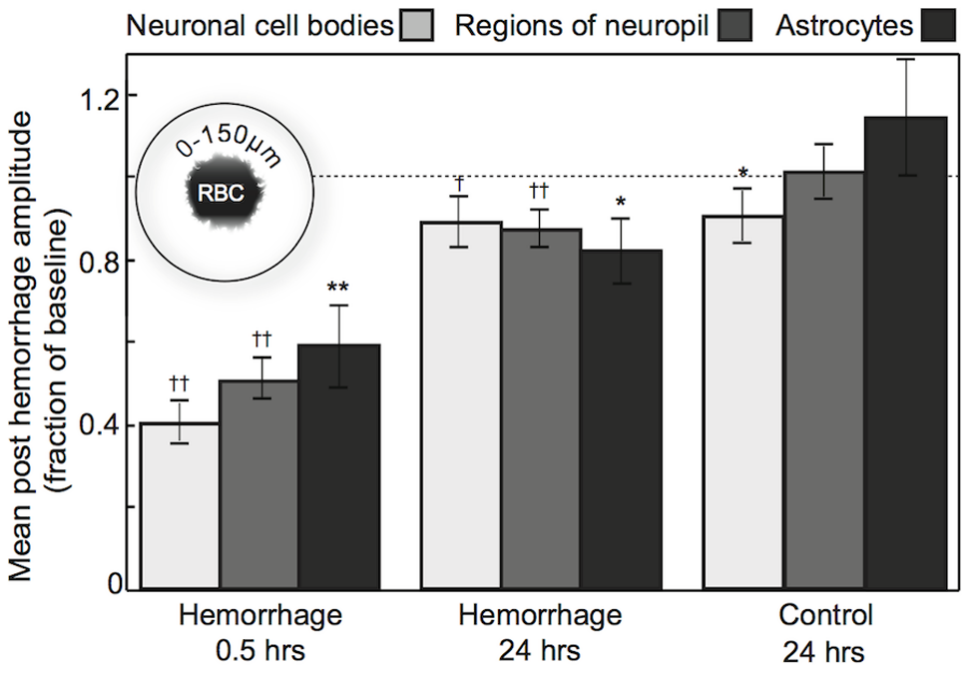
Changes in average neuronal response over one day after a microhemorrhage. Average of the post-hemorrhage stimulus-induced calcium response amplitudes, expressed as a fraction of the baseline response amplitudes, at 0.5 and 24 hours after a microhemorrhage across 7 mice (and for controls at 24 hours across 3 mice) for neuronal cell bodies (light grey), regions of neuropil (dark grey), and astrocytes (black) located within 150 µm of the RBC-filled hematoma, as indicated in the inset. Error bars represent the standard error of the mean. Dashed line indicates a response amplitude after the microhemorrhage that is equal to the baseline response. Levels of significance for differences of the average amplitude ratios from one are indicated by: * p<0.05, ** p<0.01, † p<0.001, †† p<0.0001 (Wilcoxon signed-rank test).

To investigate whether or not neural response within 150 µm from microhemorrhage recovers over a longer time, we developed a chronic mouse preparation that enabled opening of the craniotomy for re-injection of calcium-sensitive dye (Figure 6A). Using this preparation, we characterized stimulus-evoked calcium transients 24 hours after a microhemorrhage (Figure 6B-D). Because of differences in the dye injection and the inclination of the imaging plane, we were able to successfully identify about 66% (62%) of the same neuronal cell bodies (astrocytes) one day after the microhemorrhage. Similarly, in control experiments we were able to identify 74% (66%) of the neuronal cell bodies (astrocytes) after one day. We found that responses in neuronal cell bodies, regions of neuropil, and astrocytes within 150 µm of the hematoma recover to near baseline levels by 24 hours after the lesion (Figure 7).

### Microhemorrhages did not induced apoptosis in nearby brain cells

**Figure 8:**
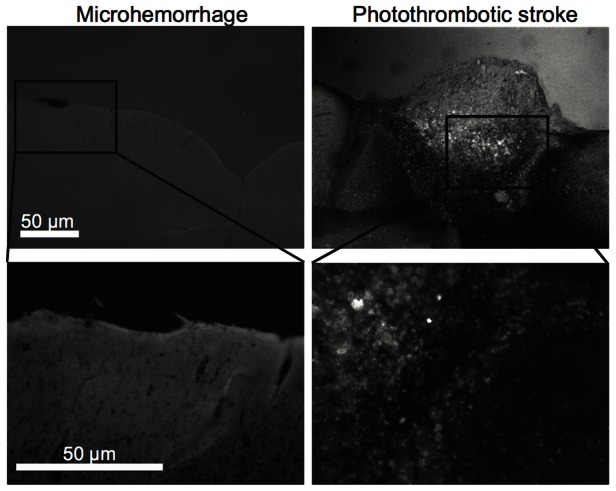
Postmortem histology of tissue near microhemorrhages. **A.** Low- and **B.** high-magnification fluorescence images for TUNEL labeling of a representative tissue section containing a microhemorrhage near the brain surface. RBCs are stained with DAB). No TUNEL-positive cells were found across 7 microhemorrhages. **C.** Low- and **D.** high-magnification fluorescence images for TUNEL labeling of a tissue section containing a larger photothrombotic ischemic lesion. TUNEL-positive cells are visible.

One possible explanation for finding only about two-thirds of the cells after the second calcium dye labeling in the chronic mouse experiments is cell death. To rule this out, we checked for apoptotic cells in the vicinity of the microhemorrhage. Across three mice, 12 microhemorrhages were produced in the cortex and seven of these were successfully identified in tissue sections collected three days after the lesion. We found no TUNEL-positive cells near the microhemorrhages (nor in any other cortical sections) (Figure 8A and Figure 8B). As a positive control, we included a section of mouse intestine with each batch of TUNEL staining and consistently observed apopototic epithelial cells (data not shown). In addition, we observed extensive TUNEL-positive cells in the penumbral region of a photothrombotic stroke (Figure 8C and Figure 8D) [24].

## Discussion

To help elucidate the mechanistic links between small brain bleeds and neurological dysfunction, we used tightly-focused femtosecond laser pulses to rupture the wall of targeted arterioles located beneath the surface of the brain in rodents to model cortical microhemorrhages [14]. We used *in vivo* nonlinear microscopy to study changes in peripheral stimulus-induced calcium transients in individual neuronal cell bodies, regions of neuropil, and astrocytes due to these microhemorrhages. We showed that neural and astrocyte function is interrupted near the microhemorrhage, but recovers over time after the lesion. Recovery occurred in a distance-dependent manner, with regions closest to the hematoma taking the longest to regain function. These studies provide the first insight into the spatial extent and time course of cortical dysfunction (and recovery) after brain microhemorrhage.

In the acute experiments in rats, we followed changes in the response of each individual neuronal cell body, region of neuropil, and astrocyte up to four hours after the microhemorrhage. For the chronic experiments in mice, however, we were only able to identify about two-thirds of the same cells on the second day of imaging, leaving open the possibility that some cells died as a result of the lesion. However, our data suggest this is unlikely because we were able to identify approximately the same number of cells on the second day of imaging in control experiments as after a microhemorrhage. Several factors may contribute to our inability to identify all of the same cells on the second day of imaging. First, the dye injection locations varied slightly between days, potentially leading to differences in which cells were labeled. Second, activated microglia and other inflammatory cells are present in the vicinity of the microhemorrhage one day after the lesion and these cells may either scavenge the calcium dye or otherwise interfere with the bulk loading process. Finally, although we consistently identified the same region of brain tissue, we often found small differences in the inclination of the imaging plane due to differences in the mouse position in the stereotaxic device between the two days of imaging. This difference in inclination leads to different cells intersecting the image plane at the edges of the frame, reducing our ability to find the same cells on both days. Further, we found no evidence of apoptotic cells in the vicinity of the microhemorrhage using histology. To give time for cells potentially affected by the microhemorrhage to progress along the apoptotic pathway and thus register as TUNEL positive, the staining was conducted at three days after the lesion. Taken together, this data suggest that the recovery in cell response seen one day after the microhemorrhage is not due to the death of the lowest-responding cells and preservation of less affected ones, but rather due to most cells recovering the ability to respond to a peripheral stimulus within one day after the microhemorrhage.

The impact of bleeding from a brain blood vessel on neural health and function depends critically on the size of the hematoma that is formed. Clinically, much of the focus has been on hematomas of a few millimeters in diameter (microbleeds) and larger. The large intracerebral hemorrhages that result in acute neurological symptoms lead to widespread cell death and are associated with high rates of mortality and morbidity in patients [25], [26]. This cell death is through to be caused by several factors, including elevations in intracerebral pressure that could cause direct cell injury and crush blood vessels causing ischemia [26], excitotoxic damage from the high levels of glutamate or neurotoxic effects of other blood products, such as thrombin, that enter the brain parenchyma [26]–[29], negative effects of the inflammation triggered by the hemorrhage [30]–[32], and damage from reactive oxygen species, such as those released by lysed red blood cells a few days after the lesion [26], [29]. On the other hand, the impact of the acutely asymptomatic microbleeds identified in MRI imaging in patients remains controversial, with some studies reporting minimal long-term cognitive effects [33], [34], and others [8], [9], [35] finding an association between microbleeds and cognitive dysfunction. Microbleeds are a frequent consequence of cerebral amyloid angiopathy, and it has been suggested that they may contribute to the cognitive decline in Alzheimer's patients [4], [9], [36], [37]. Additionally, the presence of brain microbleeds has been associated with more severe cognitive impacts after head trauma [38]. For these smaller bleeds, many of the mechanisms that lead to cell injury for large hematomas may not play a significant role. For example, there is not a significant increase in intracranial pressure. Post-mortem studies of microbleeds in humans have revealed significant inflammation near the lesion [9], [39], [40]. While there are limited studies of necrotic or apoptotic cell death after microbleeds, the current data suggest that cell death is not widespread.

Even smaller microhemorrhages that are not detectable by modern MRI likely occur in many of the same conditions as MRI-visible microbleeds. However, because these microhemorrhages (diameter <200 µm) cannot be studied in patients except through post-mortem pathology, there are fewer clinical studies available and both the cognitive and local cellular impact of these lesions remain unclear. Microhemorrahges have been found in advanced Alzheimer's disease patients and are linked with more severe cognitive problems [4], [9]. In patients with CADASIL, there is clear evidence of cognitive dysfunction [41], although multiple different kinds of brain pathologies in addition to microhemorrhages, such as lacunar infarcts and diffuse white matter changes, are found in these patients [39]. There are signs of inflammation near the lesion, including activation of astrocytes and microglia [11] and invasion of neutrophils and macrophages [9], [39], [40], but no reports have shown evidence of cell death, although there are only limited studies of these small lesions to date. In our recent work, we found that the dendrites from Layer V pyramidal neurons remained healthy and intact right up to the hematoma core [15]. We did observe a rapidly activated microglia response that led to sustained presence of inflammatory cells near the hematoma. For these small hemorrhages, it is likely that the amount of blood that enters the brain is small enough that phagocytotic cells and other mechanisms can clean the cellular environment before significant injury is caused. The work we present here suggests that these microhemorrhages do, however, lead to a transient loss of cortical function within a few hundred micrometers from the lesion, but that this function largely recovers over time.

One possibility is that the transient loss of neural function we observed is caused by the infusion of blood plasma into the intracellular space in the brain. This leads to changes in ion balances and extracellular messengers and to increases in reactive oxygen species, all of which may interfere with the ability of neurons to normally receive synaptic input and fire action potentials. Interestingly, the few hundred micrometer spatial extent of the most severe disruption of cortical function we observed correlates well with the distance that blood plasma penetrates into the parenchymal tissue in this microhemorrhage model. The less severe impact and more rapid recovery of cortical function in regions further from the microhemorrhage is consistent with a gradient in concentration of blood plasma products that cause dysfunction in the brain tissue.

The microglial activation we observed around the microhemorrhage in previous work [15] may also be important for the neural dysfunction we observe. The region of microglial activation after a microhemorrhage closely matches the spatial extent of the neural dysfunction. Microglia are thought to play a role in the normal regulation of dendritic spine morphology and dynamics [42], [43]. In addition, recent studies have suggested that after injury, activated microglia may abnormally phagocytose spines [44], likely affecting neural function. This suggests that the microglial activation we observe after a microhemorrhage may lead to changes in neural wiring that impacts function.

In this study, we did not investigate the impact of the microhemorrhage on the few neurons that were immediately adjacent to the target vessel (within ∼50 µm). These cells were likely displaced by the expanding hematoma or surrounded by red blood cells. Measurement of calcium transients from these cells was not easily achieved and so we cannot rule out the possibility that these cells are permanently dysfunctional. The lack of apoptotic cells in our histology study, however, suggests that these cells do not die, at least within three days. We also cannot rule out the possibility that lysis of red blood cells a few days after the microhemorrahge leads to additional injury that could compromise cell function later.

The short-term loss of cortical function we observed here may not be completely benign. Neurons with decreased input and firing rates may gain and lose synapses at an elevated rate due to homeostatic mechanisms that seek to maintain neural input and firing rate, thereby causing potentially aberrant rewiring of neural circuits. In addition, these hemorrhages may have a more severe impact on neural health and function if combined with disease backgrounds such as hypertension, Alzheimer's disease, or if they occur in aged brain. The microhemorrhages we studied were all in cortex, and these lesions may have a different effect in other brain locations.

Recent data has suggested that occlusion of single penetrating arterioles (the same class of vessel that we targeted to produce microhemorrhages) leads to dramatic degeneration of Layer V pyramidal cell dendrites near the ischemic core [13], [15] and to cell death over a several hundred micrometer region that can lead to measurable cognitive performance decreases [45]. In addition, although neural structures remained intact further away, there was, at least, an acute loss of peripheral stimulus-induced neural activity up to 300 µm from the lesion [13]. This data suggests that small hemorrhages have a less severe impact than occlusion of the same class of vessel, implying that clinical management of patients with cardiovascular risk factors may benefit from anticoagulation even with added risk of bleeding from small vessels.
